# Supplementary material for: Grassland productivity in response to nutrient additions and herbivory is scale-dependent
Source: PeerJ. 2016 Dec 1;4:e2745. doi: 10.7717/peerj.2745 (PMC5136131; doi:10.7717/peerj.2745)
Supplement: Table S1 — Soil nitrogen (%) and phosphorus content (mg kg−1), and soil pH (mean ± 1 standard error (SE)) across fenced treatments only, and in Mkambathi Nature Reserve, one year following nutrient additions. [file peerj-04-2745-s002.docx]

|  | **Soil N (%)** | **Soil P (mg kg^-1^)** | **Soil C (%)** | **Soil pH** | **n** |
| --- | --- | --- | --- | --- | --- |
| **Fenced, Unfertilized** | 0.166 (0.003) | 80 (3.44) | 2.5 (0.025) | 4.27 (0.025) | 33 |
| **Fenced, Homogenous** | 0.181 (0.004) | 104.9 (7.9) | 2.55 (0.013) | 4.15 (0.032) | 38 |
| **Fenced, Heterogeneous** | 0.188 (0.003) | 160 (12.5) | 2.49 (0.012) | 4.01 (0.019) | 91 |
