# Supplementary material for: Grassland productivity in response to nutrient additions and herbivory is scale-dependent
Source: PeerJ. 2016 Dec 1;4:e2745. doi: 10.7717/peerj.2745 (PMC5136131; doi:10.7717/peerj.2745)
Supplement: Table S2 — Spatial model parameters [range (3* φ), sill (σ2), nugget (τ2), and noise to signal ratio (τ2∕(τ2 + σ2)] of empirical and simulated biomass across treatments. Sill and nugget values are scaled (0–1) to facilitate comparisons across treatments. [file peerj-04-2745-s003.docx]

| **Treatment** | **Range**  **[ 3 * φ] (m)** | | **Partial Sill (scaled)**  **σ^2^ (g^2^)** | | **Nugget (scaled)**  **τ^2^ (g^2^)** | | **Nugget/sill**  **τ^2^/(τ^2^ + σ^2^)** |
| --- | --- | --- | --- | --- | --- | --- | --- |
| *Empirical* |  |  |  |  |  |  |  |
| Fenced, Unfertilized | 0.8 |  | 0.56 |  | 0.00 |  | 0.00 |
| Fenced, Homogenous | 1.9 |  | 0.31 |  | 0.00 |  | 0.00 |
| Fenced, Heterogeneous | 2.6 |  | 0.44 |  | 0.01 |  | 0.02 |
| Unfenced, Unfertilized | 17.8 |  | 0.15 |  | 0.10 |  | 0.67 |
| Unfenced, Homogenous | 12.2 |  | 0.09 |  | 0.35 |  | 3.89 |
| Unfenced, Heterogeneous | 2.6 |  | 0.16 |  | 0.00 |  | 0.00 |
| *Simulated* |  |  |  |  |  |  |  |
| Fenced, Unfertilized | 12.7 |  | 0.04 |  | 0.84 |  | 20.10 |
| Fenced, Homogenous | 12.7 |  | 0.04 |  | 0.84 |  | 20.10 |
| Fenced, Heterogeneous | 3.7 |  | 0.77 |  | 0.00 |  | 0.00 |
| Unfenced, Unfertilized | 4.5 |  | 0.76 |  | 1.00 |  | 0.10 |
| Unfenced, Homogenous | 4.5 |  | 0.76 |  | 0.07 |  | 0.10 |
| Unfenced, Heterogeneous | 4.1 |  | 0.75 |  | 0.00 |  | 0.00 |
