# Supplementary material for: Grassland productivity in response to nutrient additions and herbivory is scale-dependent
Source: PeerJ. 2016 Dec 1;4:e2745. doi: 10.7717/peerj.2745 (PMC5136131; doi:10.7717/peerj.2745)
Supplement: Table S3 — Parameters of the empirical spatial model of vegetation phosphorus and nitrogen (%), soil phosphorus (P) and nitrogen (N), and soil (C) using maximum likelihood analysis across experimental plots in Mkambathi Nature Reserve, one year following nutrient fertilization. nd=not defined. [file peerj-04-2745-s004.docx]

| **Treatment** | **Range**  **[ 3 * φ] (m)** | | **Partial Sill (scaled)**  **σ^2^ (g^2^)** | | **Nugget (scaled)**  **τ^2^ (g^2^)** | | **Nugget/sill**  **τ^2^/(τ^2^ + σ^2^)** | |
| --- | --- | --- | --- | --- | --- | --- | --- | --- |
| *Foliar N (%)* |  |  |  |  |  |  |  | |
| Fenced, Unfertilized | 1.40 |  | 0.30 |  | 0 |  | 0 | |
| Fenced, Homogeneous | 0.80 |  | 0.29 |  | 0 |  | 0 | |
| Fenced, Heterogeneous | 5.80 |  | 0.22 |  | 0.57 |  | 2.81 | |
| Unfenced, Unfertilized | 0.00 |  | 0 |  | 0.20 |  | nd | |
| Unfenced, Homogeneous | 1.30 |  | 0.51 |  | 0.05 |  | 0.09 | |
| Unfenced, Heterogeneous | 18.90 |  | 0.14 |  | 0.41 |  | 3.09 | |
| *Foliar P (%)* |  |  |  |  |  |  |  | |
| Fenced, Unfertilized | 1.50 |  | 0.26 |  | 0 |  | 0 | |
| Fenced, Homogeneous | 0.80 |  | 0.19 |  | 0 |  | 0 | |
| Fenced, Heterogeneous | 4.90 |  | 0.03 |  | 0.21 |  | 5.58 | |
| Unfenced, Unfertilized | 2.60 |  | 0.11 |  | 0.91 |  | 8.12 | |
| Unfenced, Homogeneous | 3.10 |  | 0.20 |  | 0.25 |  | 1.23 | |
| Unfenced, Heterogeneous | 113.80 |  | 0.15 |  | 0.51 |  | 3.46 | |
| *Soil N (%)* |  |  |  |  |  |  |  | |
| Fenced, Unfertilized | 40.4 |  | 0 |  | 0.08 |  | 0.26 | |
| Fenced, Homogeneous | 2.20 |  | 0.20 |  | 0.19 |  | 0.95 | |
| Fenced, Heterogeneous | 0.00 |  | 0 |  | 0.43 |  | nd | |
| *Soil P (%)* |  |  |  |  |  |  |  |  |
| Fenced, Unfertilized | 3.00 |  | 0.29 |  | 0 |  | 0 |  |
| Fenced, Homogeneous | 1.00 |  | 0 |  | 0 |  | 0 |  |
| Fenced, Heterogeneous | 0.00 |  | 0 |  | 0.52 |  | nd |  |
| *Soil C (%)* |  |  |  |  |  |  |  |  |
| Fenced, Unfertilized | 14.70 |  | 0.14 |  | 0.15 |  | 1.07 |  |
| Fenced, Homogeneous | 0 |  | 0 |  | 0.40 |  | nd |  |
| Fenced, Heterogeneous | 0 |  | 0 |  | 0.56 |  | nd |  |
