# Supplementary figures and images for: Grassland productivity in response to nutrient additions and herbivory is scale-dependent
Source: PeerJ. 2016 Dec 1;4:e2745. doi: 10.7717/peerj.2745 (PMC5136131; doi:10.7717/peerj.2745)

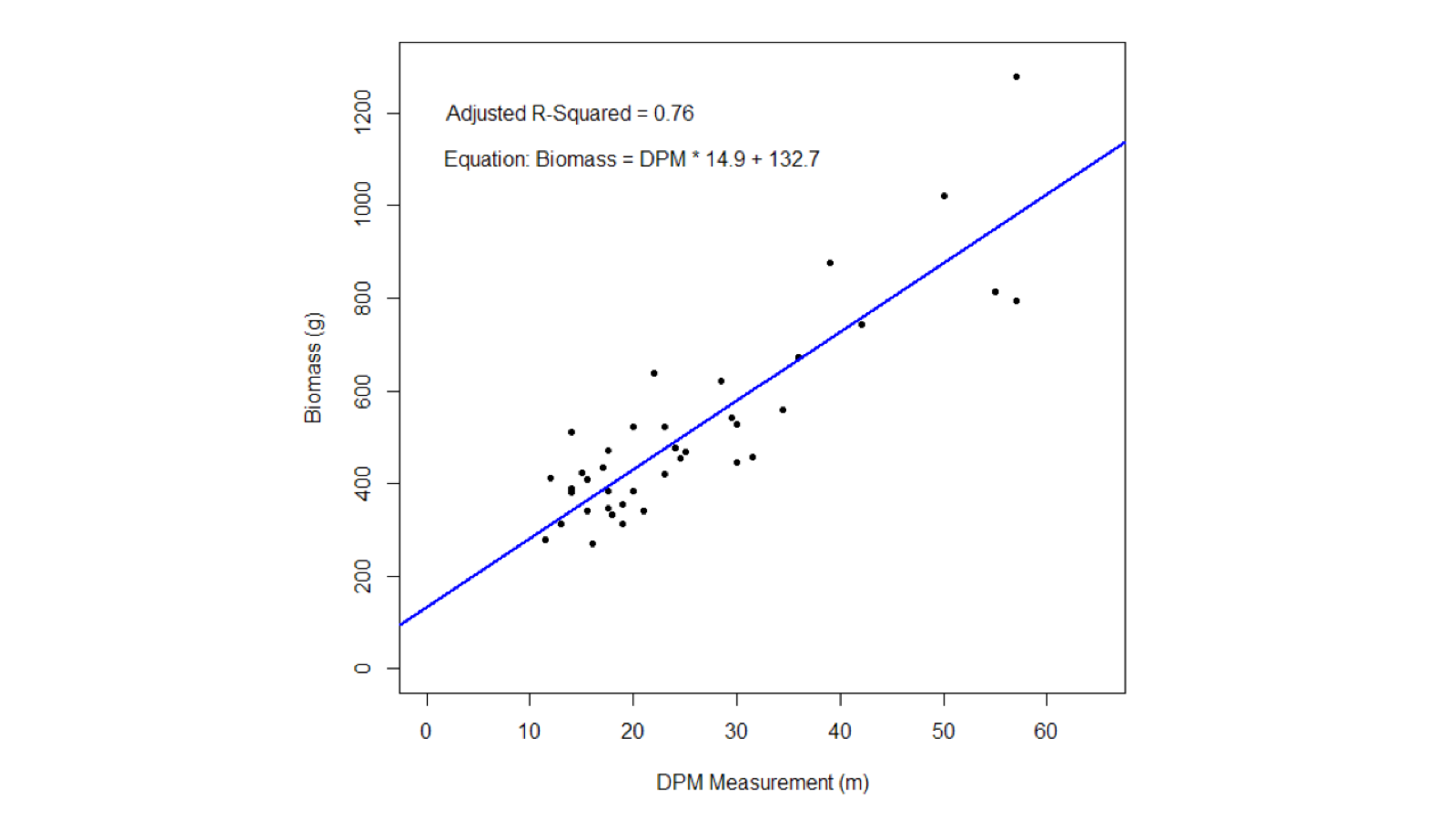

Supplement: Figure S1 — Results of the linear regression to calibrate disc pasture meter (DPM) readings to vegetation biomass (n = 60). [file peerj-04-2745-s005.png]
